# An End-to-End Trajectory Prediction Method for Unmanned Ground Vehicles via Multimodal Fusion

**DOI:** 10.3390/s26144648

**Published:** 2026-07-22

**Authors:** Yufeng Li, Erming Tian, Fuhe Yang, Huiyan Han, Xinya Zhang

**Affiliations:** 1 Shanxi Key Laboratory of Machine Vision and Virtual Reality, North University of China, Taiyuan 030051, China; 20050621@nuc.edu.cn (E.T.); 19990231@nuc.edu.cn (F.Y.); 20050537@nuc.edu.cn (H.H.); sz202415063@st.nuc.edu.cn (X.Z.); 2 Institute for Intelligent Weapon Research, North University of China, Taiyuan 030051, China

**Keywords:** unmanned ground vehicles (UGVs), multimodal fusion, end-to-end, trajectory prediction

## Abstract

To enhance unmanned ground vehicle (UGV) intelligence in smart cities, disaster rescue, and infrastructure inspection, this paper investigates the collaborative optimization of multimodal fusion end-to-end architectures. Through dynamic alignment of heterogeneous features, a multi-head distillation attention mechanism, and parallel decision-planning, a high-precision, low-latency closed-loop autonomous navigation framework is constructed. A Multi-Head Distillation Attention-based Trajectory Prediction (MDA-TP) method is proposed, combining a BEVFormer-based multimodal fusion perception model with a two-stage progressive knowledge distillation framework. On NuScenes, the method achieves an ADE of 0.88 m, an FDE of 1.32 m (4.34% and 10.81% reductions), and a collision rate of 15.2%, with 42.6 M parameters and 46 ms latency. Ablation shows removing attention distillation increases FDE by 13.6%. For system validation, a multi-sensor UGV platform is built. Through NuScenes online testing and real-world closed-loop validation, the Euclidean deviation remains within 0.5 m. Compared with traditional distillation, speed prediction MSE is reduced by 51.5%, wheel angle RMSE by 58.4%, and route completion improves from 60.99% to 97.26%. The results provide practical support for autonomous driving in smart cities, disaster rescue, and infrastructure inspection.

## 1. Introduction

The intelligence level of UGVs has become a key factor in mission effectiveness for smart cities, disaster rescue, and infrastructure inspection. According to the IFR World Robotics 2024 Report, more than 50 countries have included UGVs in their core equipment lists, with investment growing 280% over five years. Autonomous navigation in complex urban and unstructured environments ranks as the top priority for technological breakthroughs [1].

However, existing unmanned systems generally suffer from three major technical bottlenecks:

- Inefficiency of multimodal perception fusion. Cascaded fusion architectures suffer from high feature alignment error rates due to sensor heterogeneity. Under adverse conditions such as fog, night, or rain, single-modal sensor failure can cause cascading decision errors, severely limiting situational awareness [2].
- Significant decision latency of modular architectures. Sequential transmission in decoupled pipelines results in decision latency of 300–800 ms, exceeding the millisecond-level requirements for sudden obstacle avoidance. This cascaded latency has been shown to significantly increase the probability of emergency braking failure in urban tests [3].
- Weak generalization in dynamic scenes. Rule-based algorithms using pre-built maps cannot adapt to real-time environmental changes in urban and disaster scenarios (e.g., debris-filled passages, submerged roads, construction diversions) [4]. The task failure rate of UGVs in unstructured environments is more than five times higher than in structured environments.

To address these challenges, this paper explores a full-process intelligent framework integrating multimodal fusion and end-to-end architectures for UGVs.

The main contributions of this paper are as follows:

- A cross-attention-based multimodal fusion model is constructed. BEVFormer generates BEV features from images, while a dynamic hierarchical sparse 3D convolutional network extracts LiDAR features. A Transformer aligns these features to form a global environmental representation.
- An end-to-end trajectory prediction and planning network is designed. A multi-head distillation attention mechanism dynamically assigns modality-specific weights. Teacher-student pre-training with progressive distillation improves long-horizon prediction, while vectorized scene modeling optimizes decision-making paths.
- Experimental validation is conducted on a UGV platform. Joint training optimizes model parameters, with multi-dimensional evaluation covering perception accuracy, trajectory prediction, and system safety. The cross-modal multi-head attention distillation mechanism demonstrates clear advantages over traditional feature concatenation.

The remainder of this paper is organized as follows: Section 2 reviews related works on multimodal data fusion perception and end-to-end autonomous driving technology. Section 3 details the proposed materials and methods. Section 4 presents the experiments and results. Finally, Section 5 concludes the paper and outlines directions for future work.

## 2. Related Work

### 2.1. Multimodal Data Fusion Perception

Multimodal perception architectures for open-domain scenarios exhibit significant diversity in technical approaches [5]. Based on the topological structure of perception modalities, current mainstream paradigms can be divided into heterogeneous multimodal perception and unimodal visual perception. According to the fusion stage, multimodal fusion strategies are primarily categorized into early fusion (data-level), feature-level fusion, and late fusion (decision-level) [6].

Early fusion achieves low-level integration through spatial alignment of point clouds and RGB images. Feature-level fusion employs weighted feature alignment mechanisms [7]. Late fusion uses multi-branch parallel processing and model cascade confidence weighting, with methods such as CLOCs effectively improving detection confidence at the cost of increased computational load [8].

Late fusion offers flexible decoupling but suffers from geometric conversion errors and rule-based interface limitations. Early fusion better preserves raw data but requires higher computational power. Feature-level fusion balances these trade-offs.

To address these shortcomings, the Bird’s-Eye-View (BEV) representation emerged. First introduced by Tesla in 2021, BEV perception transforms multi-view camera and LiDAR data into a unified coordinate system, providing a more complete foundation for trajectory prediction and planning. Researchers have also improved environmental adaptability through dynamic visible-infrared fusion [9], lightweight embedded fusion with spatiotemporal constraints [10], and weak supervision strategies that reduce annotation requirements [11]. Attention-based fusion algorithms [12] and reinforcement learning-based methods [13] have also been explored, with collaborative perception and graph fusion approaches demonstrating improved reliability in complex environments.

In summary, significant progress has been made in multimodal fusion for autonomous driving, with growing applications in civilian domains.

### 2.2. End-to-End Autonomous Driving Technology

End-to-end systems unify perception, decision-making, and control in a single deep learning model, mapping raw sensor signals directly to control commands and avoiding cumulative errors in modular architectures [14,15,16].

(1) The evolution of key autonomous driving technologies follows three main trajectories:
(2) Temporal modeling breakthroughs: NVIDIA pioneered LSTM-based end-to-end control [17]; Cambridge Wayve demonstrated generalization with rapid fine-tuning [18]; and FCN-LSTM improved prediction accuracy by 15% [19].
(3) Multimodal fusion innovations: Yang et al.’s multi-task framework achieved an NDS score of 89.3, though long-tail control oscillations remain [20].
(4) Knowledge transfer optimization: PlanKD applies knowledge distillation to motion planner compression [21].

To address LiDAR processing costs and model uncertainty, Liu et al. proposed Fast-LiDARNet with sparse convolution and hybrid evidential fusion for uncertainty estimation [22]. Zhao et al. proposed MM-StConv with multimodal inputs to address low prediction accuracy [23]. Tesla’s FSD has demonstrated end-to-end feasibility [24], and its BEV + Transformer architecture (2021) marked a paradigm shift from 2D to 3D perception. The Transformer’s self-attention mechanism enables long-range dependency modeling, extending BEV from 3D to 4D temporal space for occluded object prediction, and supports map-free driving [25]. BEV + Transformer has become the industry consensus for perception, adopted by major manufacturers such as Huawei, Li Auto, XPeng, and NIO [26]. Transformer-based end-to-end algorithms, with their concise architecture and powerful representation, are becoming the core direction in autonomous driving [27].

Recent work has advanced end-to-end driving through FusionAD (camera-LiDAR fusion) [28], Hydra-MDP (multi-target distillation) [29], VAD (vectorized scene representation) [30], and VADv2 (probabilistic planning) [31]. Coelho and Oliveira systematically compared end-to-end methods on CARLA benchmarks [32], noting that multimodal inputs (especially camera-LiDAR fusion) and waypoint outputs are key directions for improving performance.

FusionAD first unified camera and LiDAR fusion for prediction and planning but did not address model compression or knowledge distillation. Hydra-MDP adopted teacher-student distillation, but its objective was to learn diverse trajectory candidates for different evaluation metrics. VAD introduced vectorized scene representation but lacked distillation. In contrast, our method introduces a multi-head distillation attention mechanism that performs knowledge distillation at the attention-feature level, transferring the teacher’s attention patterns to the student. Unlike Hydra-MDP’s single-stage distillation, we employ a two-stage progressive distillation framework with dynamic head weight allocation. Furthermore, we introduce a cross-modal projection layer that aligns the student’s BEV feature responses with the teacher’s, preserving multimodal feature representation capability under severe parameter constraints. These innovations achieve a 66% parameter reduction while maintaining competitive prediction accuracy. In summary, fusing multimodal sensors, introducing attention-level distillation with dynamic weighting, and adopting vectorized scene representation within a progressive distillation framework are effective ways to further enhance the performance and efficiency of end-to-end autonomous driving systems.

## 3. Materials and Methods

### 3.1. Design of the Multimodal Fusion Network Model

#### 3.1.1. BEV Encoder and Perception

**Figure 1:**
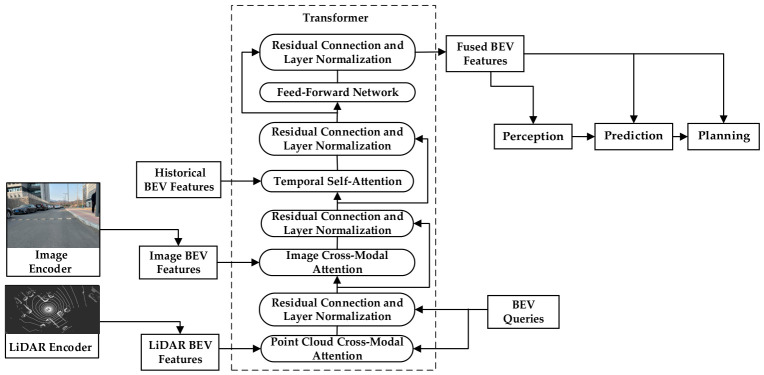
Overall architecture of the proposed multimodal fusion network. The dashed bounding box denotes the core Transformer model.

A Transformer-based multimodal fusion network model is constructed, together with a fusion-aided modality-aware prediction and state-aware planning module. On the one hand, this module integrates modality self-attention and a refinement mechanism for prediction tasks, enabling more accurate estimation of the future states of the surrounding environment and dynamic obstacles. On the other hand, for planning tasks, it introduces a collision loss and a fusion strategy with vectorized ego-vehicle information, which facilitates the generation of safer and more reasonable path planning schemes. The overall architecture is illustrated in Figure 1.

The framework adopts a hierarchical multimodal spatiotemporal architecture. A BEVFormer encoder maps camera images to BEV space, aligned with LiDAR features via coordinate system alignment. A dual-path cross-attention mechanism combines point cloud features and RGB semantics. After six layers of progressive spatiotemporal encoding, the system outputs a fused BEV representation encoding cross-modal geometric-semantic consistency and temporal motion patterns. This feeds into the modality-aware prediction and state-aware planning module.

#### 3.1.2. Point Cross-Attention Mechanism

Each BEV query interacts with LiDAR BEV features in its reference point neighborhood via deformable attention, as shown in Equation (1).

(1) $$PCA(Q_{p},B_{LiDAR})=Def\;Attn(Q_{p}{,P,B}_{LiDAR})$$

here, $Q_{p}$ denotes the BEV query at point p=(x,y), $B_{LiDAR}$ represents the BEV feature output from the LiDAR branch, and *P* is the projection of the coordinate p=(x,y) in the BEV space onto the LiDAR BEV space.

#### 3.1.3. Image Cross-Attention

To realize image cross-attention, each BEV query is extended along the height dimension, similar to the pillar representation. Within each pillar, a fixed number of $N_{ref}3D$ reference points are sampled along its Z-axis. The image cross-attention process is formulated as shown in Equation (2):

(2) $$ICA(Q_{p},F)=\frac{1}{V_{hit}}\sum_{i=1}^{V_{hit}}\sum_{j=1}^{N_{ref}}DefAttn(Q_{p},P(p,i,j),F_{i})$$

where $V_{hit}$ denotes the number of camera views to which the reference point can be projected, i is the index of the camera view, $F_{i}$ represents the image feature of the *i*-th camera, and P(p,i,j) denotes the projection of the 3D reference point $\left(x,y,z_{i}\right)$ corresponding to the BEV query $Q_{p}$ onto the image coordinate system of the *i*-th camera.

#### 3.1.4. Temporal Self-Attention

Historical BEV features are temporally aligned based on UGV motion and fused via temporal self-attention, as shown in Equation (3).

(3) $$TSA(Q_{p},\left\{Q,B_{t-1}^{\prime }\right\})=\sum_{V\in \left\{Q,B_{t-1}^{\prime }\right\}}DeformAttn(Q_{p},p,V)$$

where $B_{t-1}^{\prime }$ denotes the temporally aligned BEV features at timestamp t−1.

### 3.2. Multi-Head Distillation Attention Mechanism Architecture

#### 3.2.1. Teacher Model Architecture

The teacher model comprises vision and LiDAR branches. The vision branch uses BEVFormer to project multi-camera features into BEV space via deformable attention. The LiDAR branch uses sparse 3D convolutions to extract long-range obstacle features, compressed into a 2D BEV grid.

**Figure 2:**
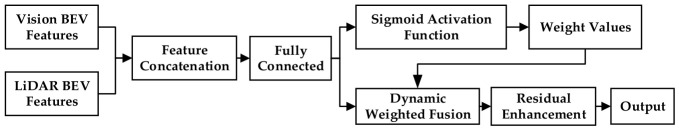
Flowchart of the multimodal feature fusion layer.

First, the spatially aligned vision BEV features (with number of channels $C_{cam}$) and LiDAR BEV features (with number of channels $C_{lidar}$) are concatenated along the channel dimension to obtain the fused feature $X_{concat}\in R^{(C_{cam}+C_{lidar})\times H\times W}$. Then, cross-modal interaction is performed to generate feature importance weights, and a gating mechanism is used to dynamically adjust the contribution weights of each modality, as shown in Equations (4) and (5):

(4) $$G=\sigma \left(Conv\left(X_{concat}\right)\right)$$

(5) $$X_{fusion}=G⊗X_{cam}+\left(1-G\right)⊗X_{lidar}$$

here, *σ* denotes the sigmoid function, and ⊗ denotes element-wise multiplication. Figure 2 illustrates the workflow of the fusion layer.

**Figure 3:**
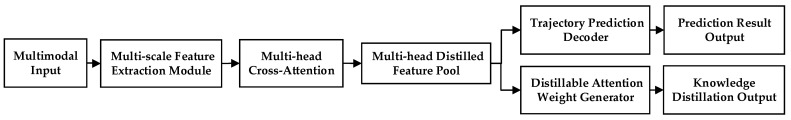
Block diagram of the teacher model.

The spatiotemporal Transformer encoder uses eight-head self-attention, with heads specialized per Table 1. Temporal information is injected via positional encoding. The encoder output feeds a multimodal prediction head that parameterizes future trajectories as a Gaussian mixture model (mean, covariance, and probability weights). The teacher model architecture is shown in Figure 3.

#### 3.2.2. Lightweight Design of the Student Model

**Figure 4:**
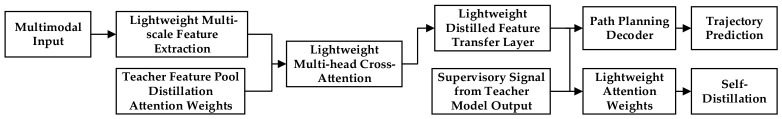
Block diagram of the lightweight student model.

The student model replaces BEVFormer with EfficientNet-B0 for vision, applies channel pruning to the LiDAR branch, and uses depthwise separable convolutions for fusion, reducing parameters to 30% of the teacher. The architecture is shown in Figure 4.

(1) Distilled Multi-Head Attention Module

The distilled attention module reduces heads from 8 to 4, compresses embedding dimensions by 50%, and shares key/value matrices across heads while retaining independent query mappings. A lightweight MLP projects student outputs to teacher space for KL divergence computation, as shown in Equation (6).

(6) $${A_{s}}^{proj}=MLP\left(A_{s}\right)\in R^{H\times T\times T}$$

This design ensures the computability of the KL divergence loss, reduces the total number of parameters in the attention layer while preserving multi-view feature representation capability, and enables more efficient spatiotemporal relationship modeling.

The four-head configuration in the student model is derived directly from the functional grouping of the teacher’s eight attention heads, as defined in Table 1. As shown in Table 1, the teacher’s attention heads are divided into four distinct functional categories: temporal continuity (heads 1–2), spatial obstacle interaction (heads 3–4), social group dynamics (heads 5–6), and cross-modal consistency verification (heads 7–8). Each student head is designed to inherit the combined knowledge of one complete functional category, preserving the four distinct specializations while reducing parameter count. This grouping is informed by the observation that attention heads within the same functional category exhibit similar attention patterns and can be effectively merged through the dynamic head weight allocation mechanism.

The decoupled key-query sharing mechanism is justified by the fact that different teacher heads primarily differ in what they attend to (queries) rather than how they encode content (keys and values). By sharing keys and values, computational cost is reduced while allowing each head to maintain its unique attention focus through independent query projections.

(2) Multi-Task Loss Function

The total loss of the student model consists of three components, as shown in Equation (7):

(7) $$L_{total}=\beta_{1}L_{pred}+\beta_{2}L_{attn}+\beta_{3}L_{feat}$$

where $L_{pred}$ is negative log-likelihood for trajectory prediction, $L_{attn}$ enforces attention consistency, and $L_{feat}$ aligns BEV feature activations. $L_{feat}$ is emphasized early in training, while $L_{attn}$ weight increases later.

### 3.3. Distillation Training and Trajectory Prediction

We adopt a lightweight multi-layer distillation scheme. By constructing a teacher-student knowledge transfer framework, the proposed method aligns the trajectory features generated by the teacher model from ground-truth data with the observed features of the student model at multiple levels (output layer/intermediate layers), thereby reducing the number of parameters while preserving prediction diversity. Specifically, a multi-head distillation attention mechanism is used to decouple the feature representations of the teacher model across different spatial scales, and a multi-sensor head decoder simultaneously transfers the logical reasoning patterns of trajectory planning. This enables the student model to generate reliable predictions even in challenging scenarios such as perceptual blind spots, ultimately achieving dual optimization of accuracy and efficiency.

#### 3.3.1. Pre-Training of the Teacher Model

The pre-training stage of the teacher model is a foundational step in constructing the distillation learning framework, with the core objective of training a high-quality knowledge source model to support subsequent knowledge transfer. Before training, multi-source heterogeneous data are processed as follows: the synchronized image sequences captured by four surround-view cameras are mapped to the BEV perspective through geometric transformation and distortion correction, generating a discretized grid representation with unified resolution. Meanwhile, LiDAR point clouds are voxelized and projected onto the same BEV coordinate system, and multimodal spatiotemporal alignment is achieved through a timestamp matching mechanism, ensuring effective joint modeling of visual semantics and geometric structure features. During training, a multimodal trajectory negative log-likelihood loss is adopted as the optimization objective. By maximizing the likelihood estimation of the observed data with respect to the parameters of a Gaussian mixture distribution, the model is guided to simultaneously capture the probability distribution characteristics of high-frequency driving trajectories (e.g., aggressive lane changes) and conservative paths (e.g., constant-speed straight driving). Model convergence is monitored using an early stopping strategy, with the Average Displacement Error (ADE) and Final Displacement Error (FDE) on the validation set as key evaluation metrics. Training is terminated when neither metric shows a significant decrease for five consecutive epochs. This mechanism ensures sufficient convergence of the model while avoiding the risk of overfitting. The teacher model was pre-trained for 100 epochs with an initial learning rate of 1 × 10^−4^, using a cosine annealing schedule to a minimum of 1 × 10^−6^.

#### 3.3.2. Progressive Distillation Training of the Student Model

Distillation proceeds in three stages: (1) coarse-grained feature matching, aligning student BEV activations with teacher features via MSE loss and L1 regularization; (2) fine-grained attention transfer, using KL divergence to align attention distributions at selected layers (3rd, 5th, 7th) with a penalty factor of 3; and (3) task-oriented fine-tuning, freezing feature extractors and tuning only the prediction head with heading and smoothness constraints.

The teacher model, despite its own open-loop collision rate, serves as a valuable knowledge source by providing multi-dimensional supervisory signals beyond final predictions. Specifically, the distillation process transfers: (1) spatial feature representations through feature response loss ($L_{feat}$), (2) attention patterns through attention distillation loss ($L_{attn}$), and (3) trajectory distributions through imitation learning loss ($L_{traj}$). These complementary mechanisms enable the student model to inherit the teacher’s scene understanding and critical region focus capabilities.

The optimization process employs the AdamW optimizer with weight decay 0.01, gradient clipping at 1.0, and an initial learning rate of 5 × 10^−4^, along with a two-stage adjustment strategy: a cosine annealing schedule (minimum learning rate 1 × 10^−5^) is used during the first three training epochs, which is then switched to a linear decay mode (final learning rate 3 × 10^−6^) during the fine-tuning stage. The KL divergence temperature was set to 1.0. To prevent model collapse, diverse data augmentations are injected at each training stage, including random rotation of the BEV grid (±15°) and Gaussian jitter of the LiDAR intensity channel.

#### 3.3.3. Online Trajectory Prediction

The inference process of online trajectory prediction adopts a strictly time-sequential processing architecture to ensure low-latency responses in real-time autonomous driving systems. When the sensor data stream of the UGV arrives, the system first performs cross-modal signal synchronization. RGB images captured by four surround-view cameras are bilinearly resized to a resolution of 640 × 360 and then fed into the vision encoder for feature extraction. Through a cross-view attention mechanism, the encoder generates a high-semantic 256-dimensional BEV grid with a resolution of 0.25 m per cell. Meanwhile, the LiDAR point cloud is processed by a dynamic sparse convolutional network to extract multi-scale geometric features, which are then projected onto a grid system aligned with the vision BEV space via max pooling, producing a vectorized representation of the geometric topology. The feature fusion module adopts a gated adaptive mechanism to perform channel-wise weighted fusion of visual semantic features and LiDAR geometric features, forming a multi-level BEV scene representation that encompasses obstacle semantics, velocity vectors, and road-edge structures.

**Figure 5:**
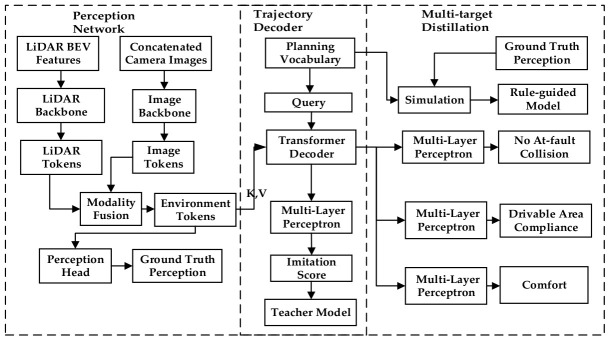
Framework of the path planning module.

In the spatiotemporal reasoning stage, the distilled and optimized four-head attention module plays a central role: each attention head constructs independent query-key matrices for different interaction dimensions (e.g., vehicle-vehicle, vehicle-pedestrian) and computes spatial correlation weights via scaled dot-product attention. The decoder stacks two layers of cross-attention networks to model the spatiotemporal correlation between the global BEV features and the historical trajectory encodings, and outputs a parameter matrix containing four Gaussian distribution modes (each mode trajectory consists of mean coordinates, a covariance matrix, and a mode weight). The trajectory generation module first produces 20 candidate trajectories, which are then filtered by a scene constraint checker to remove those that violate traffic rules or physical dynamics feasibility. The remaining trajectories are sorted by their mode probability weights, and the top-3 trajectories are selected for Bayesian fusion. Finally, the trajectory with the highest overall score, together with a visual envelope of its 95% confidence region, is output for use by the downstream planning module. The path planning process is illustrated in Figure 5.

### 3.4. Parallel Decision-Making and Planning

#### 3.4.1. Vectorized Scene Learning

To achieve safe and reliable path decision-making, autonomous driving systems require an efficient environment perception framework. Traditional modular architectures treat perception and planning as separate stages, preventing the decision-making layer from accessing the full semantics of raw sensor data. This cascaded processing mode has three major limitations: First, the planning layer fully relies on the abstract results output by the preceding perception module, missing the opportunity to correct errors using the raw sensory signals, and allowing perception errors to accumulate into systemic risks through unidirectional propagation. Second, mainstream path planning methods typically depend on high-precision rasterized maps for scene representation, which incurs high computational costs and weakens the instance-level topological relationships among traffic elements. Third, such discrete representation struggles to cope with real-time changes in dynamic environments.

This study proposes an intelligent decision-making framework based on vectorized scene understanding, which innovatively decomposes the road environment into a multimodal vector space. The core breakthroughs of the framework are as follows: by fusing BEV features, a continuous 3D vectorized scene is constructed, completely abandoning traditional raster-based discretized encoding. Meanwhile, a joint dynamic-static element embedding mechanism is introduced to convert dynamic obstacle trajectories and static road elements into explicit vector constraints, enabling real-time construction of multi-level interaction relationships such as lane topology and driving boundaries. This grid-free representation mechanism offers significant advantages in both computational efficiency and semantic fidelity.

**Figure 6:**
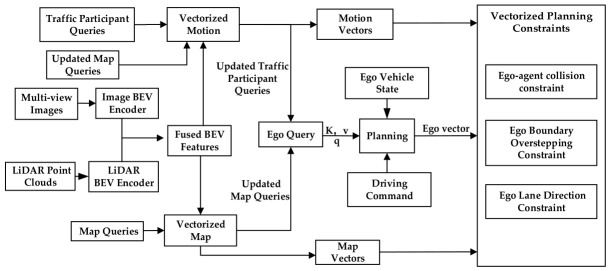
Intelligent decision-making framework based on vectorized scene understanding.

As shown in Figure 6, the system first takes multi-frame temporally fused BEV features as input. Then, a triple interaction mechanism is introduced to achieve vectorized reconstruction of scene representation. Hierarchical vector scenes are constructed through cross-attention among traffic participant queries, ego-vehicle queries, and map queries. The planning module generates future trajectory hypotheses by decoding spatiotemporally correlated features based on the interaction between ego-vehicle states and driving commands. In addition, three vectorized planning constraints are introduced to regularize the planned trajectory at the instance level, further improving trajectory safety and rationality.

(1) Vectorized Map: Map queries $Q_{m}$ extract map information from BEV features, predicting map vectors ${\hat{V}}_{m}\in R^{N_{m}\times N_{p}\times 2}$ for lane dividers, road boundaries, and pedestrian crossings, here, $N_{m}$ and $N_{p}$ denote the number of predicted map vectors and the number of points contained in each map vector, respectively.
(2) Vectorized Agent Motion: Agent queries $Q_{a}$ learn agent features via deformable attention, decoded into attributes (location, class, orientation). Agent-agent and agent-map interactions enrich motion features, producing multi-modality motion vectors ${\hat{V}}_{a}\in R^{N_{a}\times N_{k}\times T_{f}\times 2}$ with probability scores where $N_{a}$, $N_{k}$ and $T_{f}$ denote the number of predicted agents, the number of modalities, and the number of future timestamps, respectively. These constrain ego trajectory planning.

#### 3.4.2. Interactive Behavior Planning

(1) Ego-Agent Interaction

A randomly initialized ego query $Q_{ego}$ is used to learn implicit scene features valuable for the planning task. To capture the location and motion information of other dynamic traffic participants, the ego query first interacts with the agent queries $Q_{a}$ through a Transformer decoder, where the ego query serves as the q, and the agent queries serve as the Key (K) and Value (v). The ego position $p_{ego}$ and agent positions $p_{a}$ predicted by the perception module are encoded by a single-layer MLP PE_1_, serving as the query positional embedding $q_{pos}$ and key positional embedding $k_{pos}$, respectively. The above process can be formulated as:

(8) $${Q^{\prime }}_{ego}=TransformerDecoder\left(q,k,v,q_{pos},k_{pos}\right)$$

(9) $$q=Q_{ego},k=v=Q_{a}$$

(10) $$q_{pos}=PE_{1}\left(p_{ego}\right),k_{pos}=PE_{1}\left(p_{a}\right)$$

(2) Ego-Map Interaction: Updated ego query $Q_{ego}^{\prime }$ interacts with map queries $Q_{m}$ via MLP PE2 for ego and map positions as shown in Equations (11)–(13).
  (11) $${Q^{\prime \prime }}_{ego}=TransformerDecoder\left(q,k,v,q_{pos},k_{pos}\right)$$
  (12) $$q={Q^{\prime }}_{ego},k=v=Q_{m}$$
  (13) $$q_{pos}=PE_{2}\left(P_{ego}\right),k_{pos}=PE_{2}\left(P_{m}\right)$$
(3) Planning Head

The planning head takes the updated ego queries $\left(Q_{ego}^{\prime },Q_{ego}^{\prime \prime }\right)$ together with the current state $s_{ego}$ of the UGV as the ego feature $f_{ego}$, and also receives the driving command c as input, finally outputting the planned trajectory ${\hat{V}}_{ego}\in R^{T_{f}\times 2}$. This model adopts a simple MLP-based planning head. The corresponding decoding process can be formulated as shown in Equations (14) and (15):

(14) $${\hat{V}}_{ego}=PlanHead\left(f_{t}=f_{ego},cmd=c\right)$$

(15) $$f_{ego}=\left[{Q^{\prime }}_{ego},{Q^{\prime \prime }}_{ego},s_{ego}\right]$$

here, the square brackets […] denote the concatenation operation, $f_{t}$ represents the features used for decoding, and cmd represents the navigation driving command. Within the planning head, these elements are combined through concatenation and mapping to ultimately generate the planned trajectory of the UGV.

Specifically, the planning head concatenates the following elements:

- The updated ego query features $Q_{ego}^{\prime }$ and $Q_{ego}^{\prime \prime }$, which contain dynamic and static scene information obtained through interaction with agent queries and map queries.
- The current state $s_{ego}$ of the ego unmanned ground vehicle, including its position, velocity, acceleration, etc.
- The high-level driving command c, which indicates the maneuver to be executed by the vehicle (e.g., turn left, turn right, or go straight).

After concatenation, the resulting feature vector is fed into a multi-layer perceptron (MLP) for decoding, ultimately generating the planned trajectory $V_{ego}$. This process can be expressed as:

(16) $$f_{t}=Concat(Q_{ego}^{\prime },Q_{ego}^{\prime \prime },s_{ego})$$

(17) $$V_{ego}=MLP_{planning}\left(f_{t},cmd\right)$$

here, the Concat operation combines different feature vectors into a single input vector. $MLP_{planning}$ denotes the multi-layer perceptron model within the planning head, which takes the concatenated feature vector along with the driving command as input and outputs the planned trajectory $V_{ego}$. This trajectory constitutes a multimodal prediction, representing the possible motion paths of the UGV over future time steps.

#### 3.4.3. Planning Constraints and Vectorized Path Optimization

The algorithm model employs three instance-level vectorized planning constraints based on the learned map and motion vectors. During the training phase, these constraints regularize the planned trajectory, thereby improving the safety and accuracy of planning.

(1) Collision Constraint between Ego Vehicle and Other Traffic Participants

For multi-vehicle interaction scenarios, differentiated criteria for lateral tolerance and longitudinal safety margin are designed to achieve adaptive adjustment of multi-dimensional safety distances. Accordingly, different distance thresholds $\delta_{x}$ and $\delta_{y}$ are adopted for the lateral and longitudinal directions, respectively. For each future timestamp, the closest other traffic participant within a certain range $\delta_{a}$ is identified in both directions. For each direction i∈{X,Y}, if the distance dis to the closest participant is less than the threshold $\delta_{i}$, the loss term of this constraint is $\delta_{i}-dis$; otherwise, it is 0. The collision constraint loss between the ego vehicle and other traffic participants can be formulated as shown in Equations (18) and (19).

(18) $$L_{col}=\frac{1}{T_{f}}\sum_{t=1}^{T_{f}}\sum_{i}L_{col}^{it},i\in \left\{X,Y\right\}$$

(19) $$L_{col}^{it}=\left\{\begin{array}{c} \delta_{i}-d_{a}^{it},ifd_{a}^{it}<\delta_{i}\\ 0,ifd_{a}^{it}\geq \delta_{i} \end{array}\right.$$

(2) Ego-Vehicle to Road Boundary Constraint

Reliable road boundary predictions are first selected by filtering with a low-confidence threshold. For each future timestamp, the distance between the planned waypoint and its nearest map boundary line is calculated. The loss for this constraint is defined as follows:

(20) $$L_{bd}=\frac{1}{T_{f}}\sum_{t=1}^{T_{f}}L_{bd}^{t}$$

(21) $$L_{bd}^{t}=\left\{\begin{array}{c} \delta_{bd}-d_{bd}^{t},ifd_{bd}^{t}<\delta_{bd}\\ 0,ifd_{bd}^{t}\geq \delta_{bd} \end{array}\right.$$

(3) Ego-Vehicle to Lane Direction Constraint

The motion direction of the ego vehicle should be consistent with the direction of the lane in which it is located. First, the map predictions are filtered using a low-confidence threshold. Here, “map” refers to the process of converting low-confidence prediction data into specific outputs, such as the direction of the lane where the ego vehicle resides. For each future timestamp, the nearest lane divider vector is found from the planned waypoint. The loss of this constraint is defined as the time-averaged angular difference between the lane vector and the ego vehicle vector, as shown in Equation (22).

(22) $$L_{dir}=\frac{1}{T_{f}}\sum_{t=1}^{T_{f}}F_{ang}\left(v_{m}^{t},v_{ego}^{t}\right)$$

The autonomous driving planning framework is based on a vector-field cooperative constraint mechanism and adopts a dual-layer vector modeling approach. At the explicit level, a structured representation network consisting of road boundary vectors, lane centerline vectors, and dynamic obstacle trajectory vectors is constructed to accurately characterize the drivable area boundaries and the motion tendencies of traffic participants. At the implicit level, a deep attention network is employed to fuse the ego-vehicle state with environmental semantics, building a dynamic potential field to perceive driving risks.

## 4. Experiments and Results

### 4.1. Simulation Experiments

This experiment aims to validate the effectiveness of the proposed end-to-end trajectory prediction method for autonomous driving based on knowledge distillation and multi-head attention mechanisms. The core objectives include: exploring the performance advantages of combining knowledge distillation with multi-head attention; verifying the feasibility of extending the distillation supervision from the output layer to the intermediate features of the attention mechanism; and evaluating the cross-modal joint distillation effect after aligning the representation differences between visual BEV features and LiDAR BEV features through a projection layer, as well as analyzing its robustness in complex scenarios.

#### 4.1.1. Experimental Setup

In this work, the trajectory prediction and planning problem is defined as follows. Given a sequence of historical observations from multiple sensors up to time *T*, the goal is to predict the future trajectory of the ego vehicle and other traffic participants. The input consists of *L* consecutive multi-modal observations $O_{t}=\{I_{t},P_{t},V_{t}\}$, where $I_{t}\in R^{H\times W\times 3}$ denotes the multi-view RGB camera images (*H* = 360, *W* = 640, 4 cameras), $P_{t}\in R^{N\times 4}$ denotes the LiDAR point cloud (*N* points per frame), $V_{t}\in R^{D}$ denotes the ego-vehicle kinematic state (position, velocity, yaw, acceleration), and *L* = 4 frames (2 s of history at 2 Hz sampling rate). The output is a set of future trajectories $\Gamma =\{\gamma_{0},\gamma_{1},\ldots ,\gamma_{K}\}$ for *K* timestamps, where *K* = 10 (5 s prediction horizon at 0.5 s interval), with each trajectory $\gamma_{K}\in R^{2}$ as a waypoint in the global coordinate system. The internal BEV feature map is represented as $B\in R^{200\times 200\times 256}$ with a resolution of 0.25 m per cell. The total loss function is defined in Section 3.2.2.

The nuScenes autonomous driving dataset is used as the benchmark. It contains multi-view camera images, LiDAR point clouds, and corresponding high-precision trajectory annotations, making it suitable for multimodal fusion and long-horizon trajectory prediction tasks. Three categories of evaluation metrics are adopted: for trajectory prediction performance, Average Displacement Error (ADE), Final Displacement Error (FDE), and collision rate are used.

To ensure a fair comparison, four baseline models are set up: an uncompressed teacher model based on the full multimodal fusion architecture; a traditional knowledge distillation model that only supervises the output layer of the student network; a lightweight student model without distillation, trained solely with task loss; and the proposed multimodal attention distillation model.

The implementation of the models consists of two parts. The teacher network uses a visual BEV feature extractor and a LiDAR BEV feature extractor, fuses the features through an 8-layer multi-head Transformer, and predicts future trajectories for 5 s (with an interval of 0.5 s) based on the multi-head distillation attention mechanism. The student network replaces ResNet-50 with EfficientNet-B0 to compress the vision branch, halves the number of channels in the LiDAR branch, uses a 2-layer lightweight Transformer for feature fusion, and introduces a KL divergence loss to align the attention weight distributions between the teacher and the student. During training, the visual images are normalized, and the LiDAR point clouds are rasterized with a voxel resolution of 0.2 m and aligned to the BEV features. A hybrid optimization strategy is adopted for the loss function. The training batch size was set to 4 per GPU. The final trajectory data are stored in CSV format, with fields as shown in Table 2.

#### 4.1.2. Simulation Experimental Results and Analysis

(1) Visual Analysis of Predicted Trajectories

**Figure 7:**
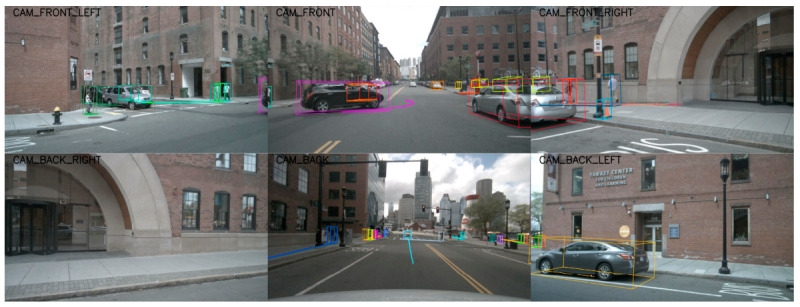
Acquired data and object detection results of the first scenario.

**Figure 8:**
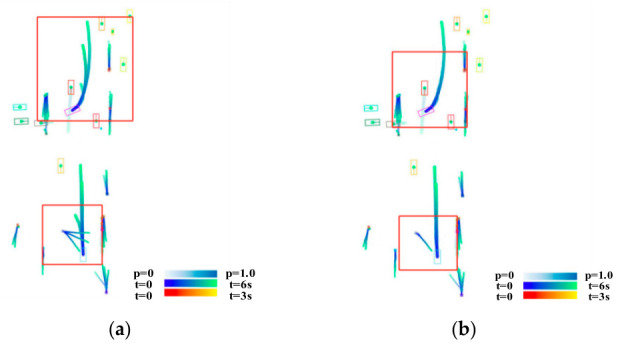
Comparison of trajectory predictions before and after distillation in the first scenario. (**a**) Trajectory prediction results before distillation; (**b**) Trajectory prediction results after distillation.

**Figure 9:**
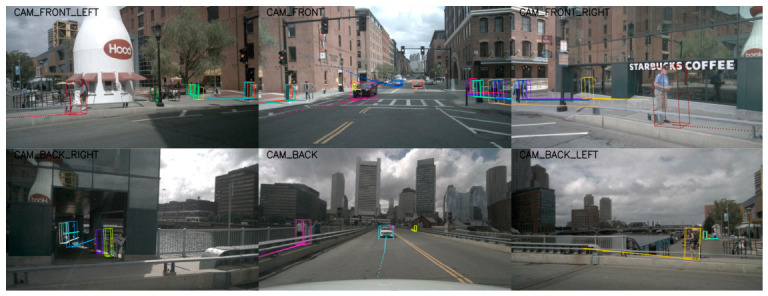
Acquired data and object detection results of the second scenario.

**Figure 10:**
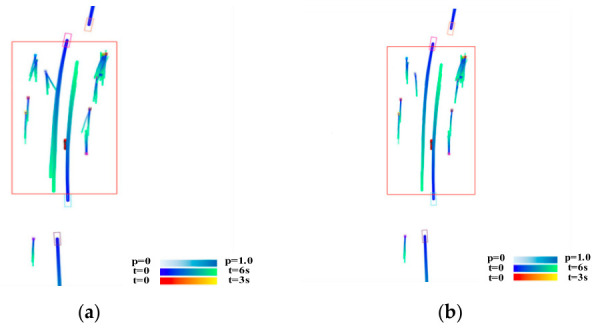
Comparison of trajectory predictions before and after distillation in the second scenario. (**a**) Trajectory prediction results before distillation; (**b**) Trajectory prediction results after distillation.

The outputs of the distilled student model are visualized and compared with those of the model without distillation. The results cover two scenarios. The first scenario involves a turning road segment, as shown in Figure 7 and Figure 8. In this scenario, the student model without distillation produces divergent trajectory predictions with unclear driving intentions and exhibits delayed responses to encroachments by adjacent vehicles. In contrast, after knowledge distillation, the model demonstrates significantly enhanced scene understanding. The multimodal trajectories converge to reasonable maneuvering regions after obstacle avoidance, with more concentrated probability distributions, clear intentions, and well-defined safety boundaries. The second scenario is a straight road, as illustrated in Figure 9 and Figure 10. In this scenario, the student model without distillation generates a large number of redundant modes when driving straight. The distilled model, by imitating the safe strategy of the teacher model, improves its sensitivity to interactive scenarios, reduces the number of output modes, and produces a more reliable intention distribution.

Next, to analyze the limitations of the student model relative to the teacher model, a qualitative evaluation of its performance is conducted.

1) Trajectory Prediction Performance Comparison

**Figure 11:**
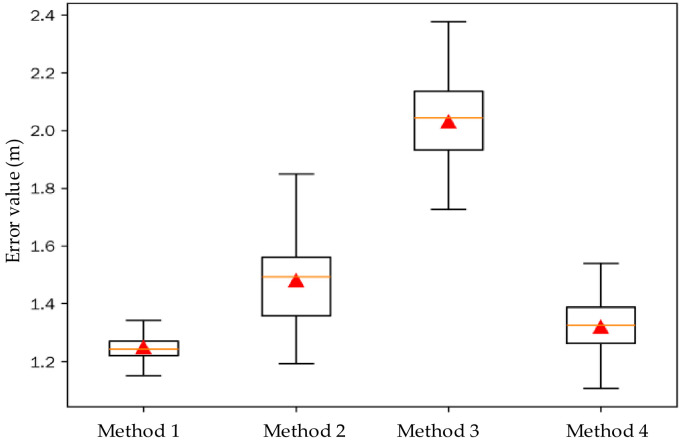
Distribution of final displacement error (FDE) for trajectory prediction.

**Figure 12:**
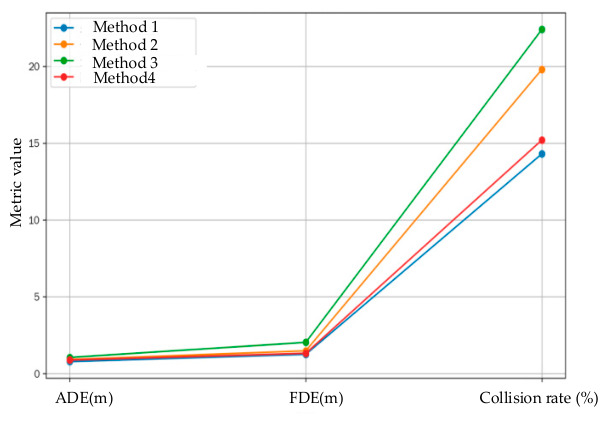
Performance comparison of different methods.

Table 3 presents the trajectory prediction results of different methods on the nuScenes test set. The proposed method achieves an ADE of 0.88 m and an FDE of 1.32 m. Compared with traditional knowledge distillation methods, the proposed approach reduces the ADE and FDE by 4.34% and 10.81%, respectively, and lowers the collision rate to 15.2%, approaching the accuracy of the teacher network within a lightweight model. As shown in Figure 11, the Final Displacement Error (FDE) is significantly reduced (Methods 1 to 4 correspond to those listed in Table 3). Figure 12 demonstrates that the proposed method achieves better long-term prediction performance, yielding more stable and reliable planning results. Although the improvement in Average Displacement Error (ADE) is relatively modest, it has practical value in suppressing high-frequency oscillations.

It is important to contextualize the reported collision rate of 15.2%. Unlike closed-loop simulation benchmarks (e.g., CARLA) where the ego vehicle continuously replans and corrects its trajectory, the nuScenes open-loop evaluation measures collisions in single-step predictions without feedback. As noted by Zhai et al. [33], this protocol can produce higher collision rates and does not fully reflect real-world planning performance. Moreover, collision rate definitions vary across studies in terms of collision-checking implementation, object filtering, and time horizon. Therefore, the key metric for validating our method is the relative improvement over baselines (a 23.2% reduction compared with traditional knowledge distillation and a 32.1% reduction compared with the lightweight student without distillation) rather than the absolute collision rate. 

To provide a fair context for evaluating our method’s competitiveness, we compare it with recent SOTA methods, including UniAD, VAD, VADv2, and FusionAD. Direct numerical comparison is challenging due to differences in evaluation protocols. Specifically: (1) FusionAD uses multi-modal inputs (camera + LiDAR) and closed-loop optimization, while our method uses camera-only input under open-loop evaluation; (2) VAD reports collision rates of 0.22–0.32% on nuScenes under open-loop evaluation with camera-only input—our 15.2% collision rate is within the same order of magnitude; and (3) differences in collision-checking implementation (occupancy map vs. oriented bounding box), time horizon, and object filtering significantly affect reported numbers. In terms of model efficiency, our method achieves 42.6 M parameters, compared to 125.6 M for FusionAD, demonstrating a clear advantage for edge deployment.

2) Computational Efficiency Comparison

Table 4 presents a comparison of computational efficiency before and after distillation.

As shown in Table 4, the uncompressed teacher model features a complete fusion framework that includes multi-level feature extraction, cross-modal interaction modules, and a trajectory prediction module, resulting in high computational complexity and the highest latency. The traditional knowledge distillation model exhibits lower computational complexity and latency and improves prediction performance through distillation loss. The lightweight student model without distillation achieves even lower computational complexity but suffers from poor prediction performance due to the absence of knowledge distillation. The proposed multimodal attention distillation model has a slightly higher computational complexity than traditional distillation methods, with only 42.6 M parameters and 32.8 G FLOPs. However, by introducing multimodal attention distillation, it significantly enhances prediction performance and is well-suited for edge computing deployment.

(2) Attention Mechanism Analysis

1) Visualization of Query Vector Q Sparsity

**Figure 13:**
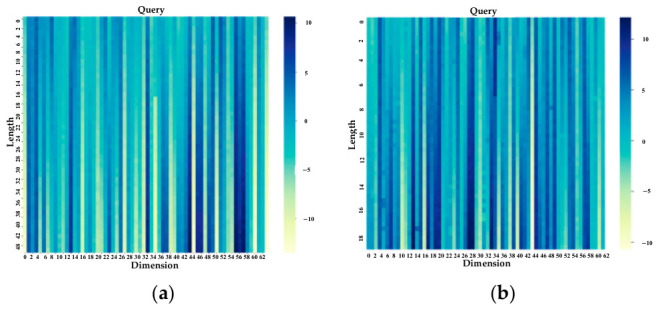
Sparsity measurement process of the query vector Q. (**a**) Distribution of the query vector Q before sparsification; (**b**) Distribution of the query vector Q after the sparsification operation.

Figure 13 illustrates the sparsity measurement process of the query vector Q. The vertical axis represents the length of the query vector, the horizontal axis represents its dimension, and the points in the figure indicate the specific values of the query vector. As shown in Figure 13a, before sparsification, the measurement distribution is uneven, with numerous near-zero points, leading to a large number of meaningless dot-product operations. To address this issue, this paper adopts a multi-head distillation attention mechanism to perform feature resampling on the query vector Q during self-attention computation, i.e., carrying out a sparse measurement process. Figure 13b shows the distribution of Q values after resampling. Compared with Figure 13a, the resampled distribution highlights more effective feature regions and contains significantly fewer near-zero points, thereby retaining more critical points, eliminating a large amount of redundant computation in dot-product operations, and improving both training and inference efficiency.

2) Multi-Head Attention Visualization

**Figure 14:**
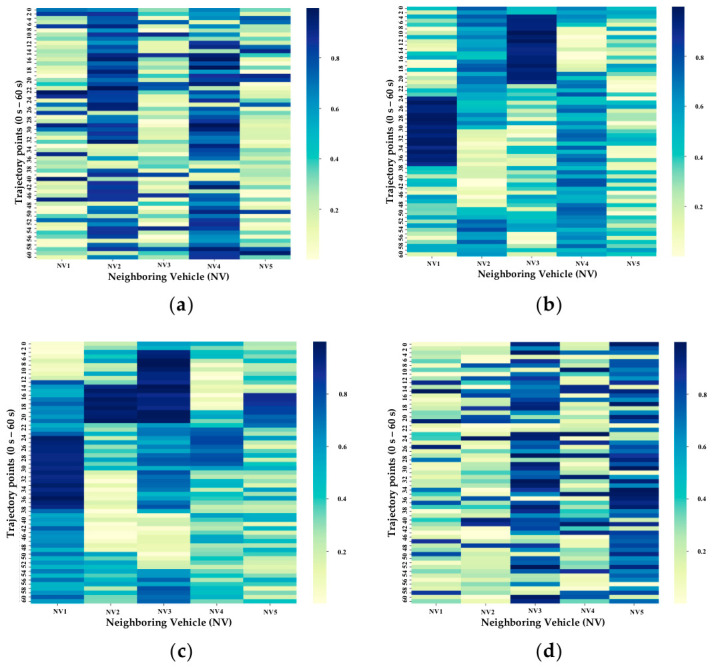
Multi-head attention distribution maps. (**a**) Attention distribution for the first selected trajectory; (**b**) Attention distribution for the second selected trajectory; (**c**) Attention distribution for the third selected trajectory; (**d**) Attention distribution for the fourth selected trajectory.

Figure 14 presents the attention values for four randomly selected trajectory samples from the test set. The vertical axis represents the trajectory points of the target vehicle (TV) from 0 to 60 s, while the horizontal axis represents five neighboring vehicles (NV1 to NV5). The color intensity ranges from light to dark, indicating attention values from low to high. The figure illustrates the different attention weights assigned to each node at each time step during the prediction network’s comprehension of the temporal multi-vehicle topological graph. In Figure 14a, NV2 and NV4 receive higher attention throughout the entire 60 s time horizon. Although other vehicles may exhibit larger attention values at certain specific moments, overall, the prediction network recognizes that NV2 and NV4 have a significant influence on the future trajectory of the target vehicle; therefore, assigning more attention to these vehicles is more meaningful. Similarly, in Figure 14b–d, the prediction algorithm allocates distinct attention values to different vehicles across different trajectories.

In traffic flow, the randomness of neighboring vehicles leads to dynamic changes in their influence on the target vehicle, resulting in continuous spatiotemporal interactions among vehicle trajectories. When processing historical multi-vehicle trajectories, the proposed algorithm not only associates the trajectories of neighboring vehicles but also dynamically captures interaction features among vehicles through an attention mechanism. For different interaction events, the model assigns differentiated attention weights to concentrate on the features of key neighboring vehicles. By fusing the target vehicle’s own trajectory with the weighted influences of neighboring vehicles, the model enhances its ability to interpret complex interaction patterns, thereby improving the accuracy and rationality of trajectory prediction.

(3) Ablation Study and Module Contribution

**Figure 15:**
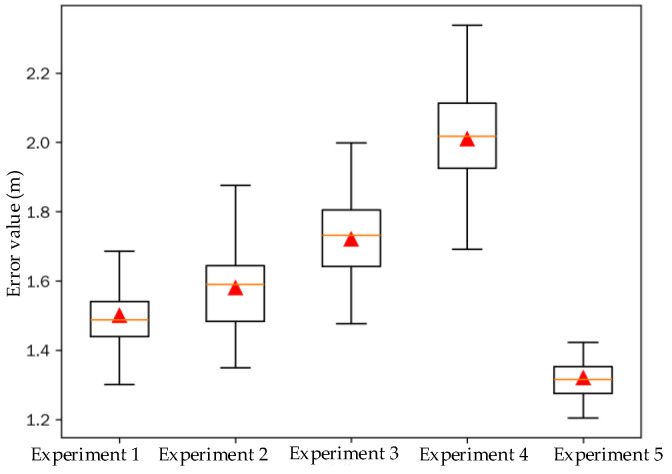
Distribution of final displacement error for trajectory prediction.

The performance of the distillation model is jointly influenced by three components of the distillation loss. To analyze the contribution of each loss component to the model performance, ablation experiments are conducted. The results are presented in Table 5, and the corresponding trajectory error distributions are shown in Figure 15.

The ablation study in Table 5 further demonstrates that the distillation process reduces both prediction error and collision risk. Removing attention distillation ($L_{attn}$) increases FDE from 1.32 m to 1.50 m and raises the collision rate from 15.2% to 19.8%, while removing both attention and feature distillation (i.e., relying solely on $L_{traj}$) increases the collision rate to 22.4%. These results indicate that the distillation process effectively transfers safety-relevant knowledge, reducing both prediction error and collision risk.

When the attention distillation loss is removed, the student model cannot learn the attention weights for critical time steps (e.g., vehicle lane changes, sudden pedestrian turns) from the teacher model, leading to a significant degradation in final displacement error (FDE). When the feature response loss is removed, the intermediate features of the student model may deviate from those of the teacher, causing unreasonable fluctuations in trajectory prediction in complex scenarios such as dense intersections. The feature response loss is crucial for global consistency and robustness; its absence results in a marked increase in average displacement error (ADE). When both the attention distillation loss and the feature response loss are removed, knowledge distillation completely fails, and the student model relies solely on the main task loss (*L_traj_*) without transferring any inductive biases from the teacher model. Supervision by *L_traj_* alone is insufficient to cover complex interaction scenarios, leading to overly conservative predictions (e.g., a bias toward straight-line driving). The complementarity among the three loss components demonstrates that joint optimization yields the best performance.

Experimental results show that the proposed method achieves trajectory prediction accuracy comparable to that of the teacher network under a lightweight framework, while significantly improving computational efficiency. The introduction of attention feature distillation and the cross-modal projection layer effectively mitigates the negative impact of multimodal representation differences on the lightweight model, exhibiting stronger robustness, particularly in extreme scenarios. The ablation study clearly quantifies the contribution of each loss component to model performance, providing a basis for subsequent optimization and laying a technical foundation for real-world onboard deployment.

### 4.2. Real-Vehicle Validation Experiments

#### 4.2.1. Experimental Environment Configuration

The hardware platform used in the experiments is the UGV equipped with four monocular cameras, a RoboSense 32-channel LiDAR (scanning frequency of 10 Hz), a millimeter-wave radar, an IMU, and a GPS module, which is employed for multimodal data acquisition and autonomous driving experiments. The monocular cameras provide RGB images with a resolution of 3840 × 2160 at 30 fps, and the LiDAR generates high-precision 3D point clouds. Multiple vision sensors are configured to enable obstacle detection, object detection, and tracking functions in autonomous driving. Through perception fusion, all targets are further processed to obtain more accurate object categories, distances, dimensions, and velocities.

Data processing and model inference are performed on a domain controller, Jetson AGX Orin 64G, which is pre-installed with the Ubuntu 20.04 LTS operating system, CUDA parallel computing platform, and cuDNN deep learning acceleration library to leverage GPU computing power. The multimodal fusion model is built on the PyTorch1.10.0 framework, and the torchvision library is used for visual data processing. Sensor data synchronization and real-time control rely on ROS. All software environments, including graphics drivers, CUDA, and cuDNN, are pre-deployed to ensure GPU acceleration efficiency during PyTorch model training and inference.

**Figure 16:**
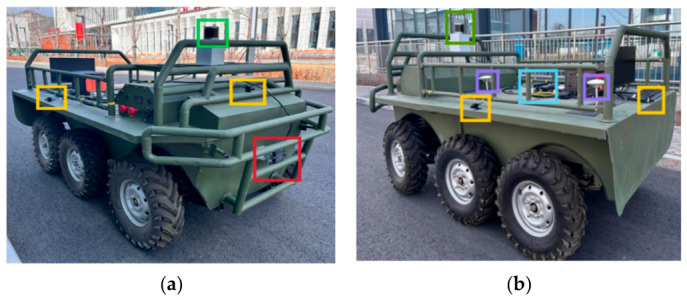
Experimental setup of the unmanned ground vehicle. (**a**) Front side view; (**b**) Rear side view.

Figure 16 shows the unmanned ground vehicle built for these experiments. The system is equipped with four-view cameras (within the yellow boxes), a LiDAR (green box), a millimeter-wave radar (red box), a navigation system (purple box), and a domain controller (blue box).

#### 4.2.2. Real-World Scene Data Collection

Real-world scene datasets are an essential requirement for the development of autonomous driving systems. By capturing full-element road data, they provide a gold standard constrained by physical laws for algorithm training and validation. This study overcomes the limitations of simulation systems in modeling interaction complexity. A two-tier scene design is adopted in the experiments: routine scenarios are used to verify basic performance metrics and robustness, while targeted scenarios focus on extreme traffic interaction cases, ensuring the system’s effectiveness and reliability under a wide range of real-world driving conditions.

(1) Routine Test Scenarios

**Figure 17:**
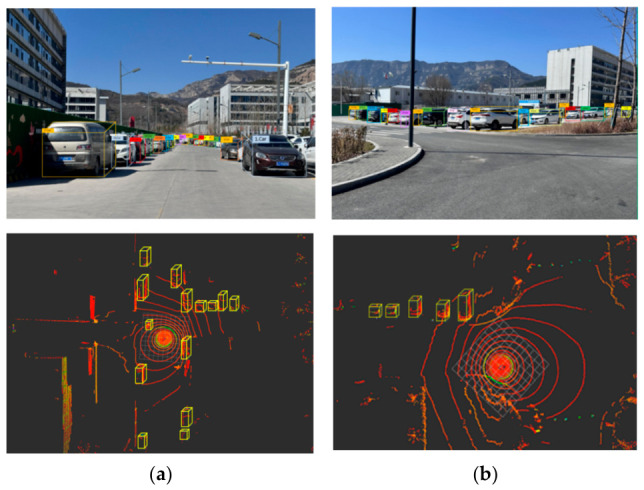
Routine test scenarios. (**a**) Straight-driving scenario; (**b**) Unprotected intersection scenario.

Routine test scenarios constitute the basic validation step in autonomous driving algorithm development. They provide a standardized and repeatable testing environment, help verify the system’s fundamental functions, assess its performance and reliability in typical driving situations, and supply data support for subsequent optimization. In this paper, the most frequent scenarios—namely, straight-driving scenarios and unprotected intersection scenarios—are designed, as shown in Figure 17.

(2) Targeted Test Scenarios

**Figure 18:**
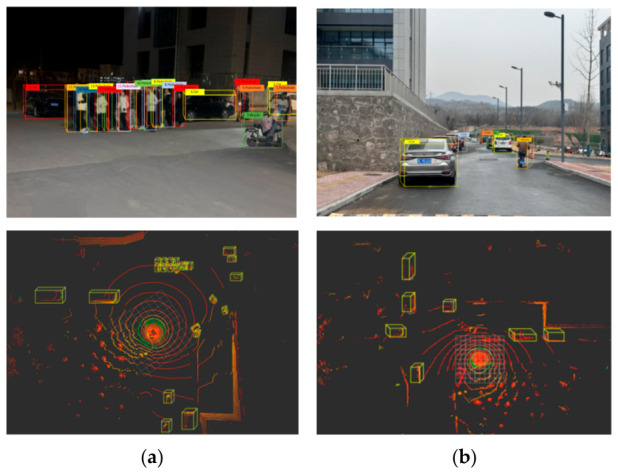

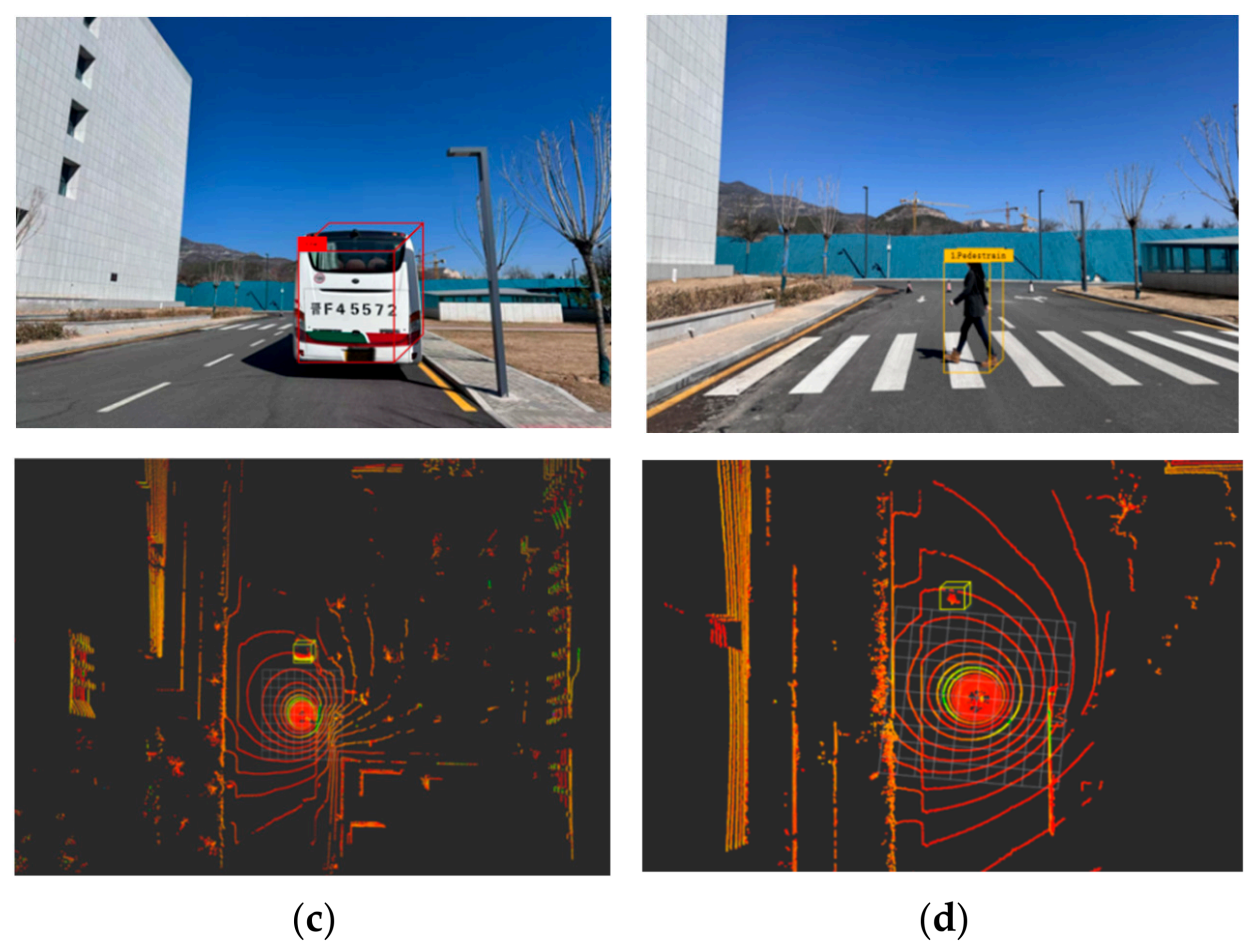
Targeted test scenarios. (**a**) Dusk scene; (**b**) Rainy scene; (**c**) Car-following scene; (**d**) Pedestrian crossing scene.

Targeted test scenarios focus on driving environments under extreme or special conditions, simulating adverse weather, nighttime conditions, dense traffic, complex intersections, and other special situations. Their main purpose is to identify potential issues and limitations of autonomous driving algorithms in real-world applications, thereby providing a basis for algorithm optimization. To this end, four scenarios are designed: a dusk scene, a rainy scene, a car-following scene, and a pedestrian crossing scene, as shown in Figure 18.

Rainy scenes cause significant interference to sensor data (especially cameras). Such scenes can be used to verify the robustness and reliability of the multimodal perception system under degraded data conditions. Dusk scenes test the system’s adaptability to low-light environments, evaluating whether it can achieve accurate perception when the performance of visual sensors deteriorates. Pedestrian crossing scenes are employed to validate the system’s trajectory prediction and decision-planning capabilities in dynamic environments, ensuring efficient operation in complex traffic flows during peak hours. Complex intersection scenarios further examine the system’s path planning and decision-making abilities under complicated road conditions, verifying its reliability in real-world applications. In summary, targeted test scenarios serve to comprehensively evaluate the performance of autonomous driving algorithms under extreme conditions, thereby improving the system’s robustness and safety.

#### 4.2.3. Experimental Setup and Parameter Tuning

**Figure 19:**
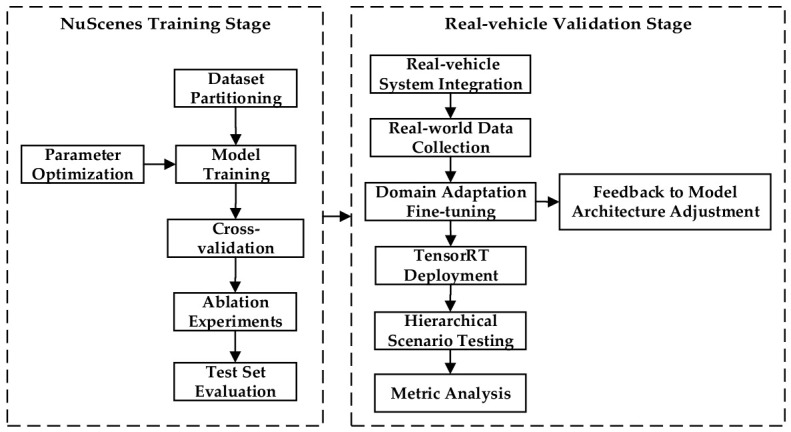
Experimental workflow.

In the experimental setup, the nuScenes dataset is first divided into training, validation, and test sets with ratios of 70%, 15%, and 15%, respectively, ensuring that nighttime and rainy scenes account for at least 15% of each subset. Subsequently, multimodal data are aligned by timestamps, sensor data are normalized, and BEV features are extracted. To ensure model generalization, a cross-validation approach is adopted for model evaluation. For the custom dataset, real-world road data collected by the unmanned ground vehicle are used for model fine-tuning and real-scene validation. The overall experimental workflow is illustrated in Figure 19.

The core objective of parameter tuning is to achieve an optimal balance among prediction accuracy, real-time performance, and robustness of the system in complex urban scenarios by adjusting model architecture, training strategies, and environment interaction parameters. A combination of grid search and random search is adopted to optimize hyperparameters such as learning rate, batch size, and fusion layer weights. In the pre-training stage, the structural parameters of the multimodal fusion module (e.g., number of attention heads, depth of fusion layers) are tuned, and Bayesian optimization is used to determine the number of Transformer layers and attention heads that minimize the validation set Average Displacement Error (ADE). In the fine-tuning stage (based on a custom dataset), the tuning focuses on controlling the cost function weights of the planning module and the intensity of data augmentation. After adjusting the path smoothness weight (from 0.7 to 0.5), the collision rate is reduced. Finally, the set of parameters that achieves the best performance on the validation set is selected for subsequent experiments.

All real-world experiments were conducted on a dedicated test track at the Shanxi Key Laboratory of Machine Vision and Virtual Reality, with the following safety provisions: (1) a rule-based safety supervisor that monitors the planned trajectory and overrides any command that would cause the vehicle to leave the drivable area or approach obstacles within 1.0 m; (2) a manual emergency stop system operated by a safety driver; (3) a speed limiter (maximum 10 km/h); and (4) redundant braking via both the planning system and a separate hardware-based emergency braking module. These mechanisms were not triggered in any of our reported test runs except during deliberately challenging edge-case scenarios.

During all reported test runs, no safety interventions (rule-based supervisor override, manual emergency stop, speed limiter activation, or redundant braking) were triggered, except in deliberately challenging edge-case scenarios. This indicates that the reported safety performance is primarily attributable to the learning model itself.

A total of 100 experimental runs were conducted across four scenario types (straight road, curved road, debris passage, and inspection park), with 25 runs per scenario. The same trained student model was deployed on the Jetson AGX Orin platform without retraining across all scenarios.

The implementation follows a two-stage training pipeline. In the perception network pre-training stage, the BEVFormer-based encoder is trained on image data with a learning rate of 1 × 10^−4^ for 100 epochs, using L1 regression loss and cross-entropy loss, with cosine annealing to 1 × 10^−6^. Joint training with the LiDAR branch runs for 500 epochs, with the BEV resolution increased from 200 × 200 to 300 × 300 at epoch 400 and the learning rate reduced to 1 × 10^−5^. Data augmentation includes random horizontal flip (0.5), brightness jitter (±10%), Gaussian noise (σ = 0.01) for images, and random rotation (±5°), global position shift (±0.5 m), and 20% random dropout for LiDAR point clouds. For real-vehicle deployment, the model is converted to TensorRT FP16 on a Jetson AGX Orin 64G, achieving 46 ms inference latency at a 20 Hz control frequency. Source code and trained weights will be publicly released upon acceptance.

### 4.3. Experimental Results and Analysis

#### 4.3.1. Evaluation Metrics and Methodology

To comprehensively evaluate the performance of the autonomous driving system, we design evaluation metrics from three dimensions: environmental perception accuracy, trajectory prediction and control metrics, and system safety. These metrics enable both quantitative and qualitative analysis of the system’s performance. The specific definitions and mathematical formulations are as follows:

(1) Environmental Perception Metrics

For the perception module, we focus on evaluating the accuracy and robustness of multimodal fusion-based perception.

Average Precision (*AP*): *AP* is adopted to measure the model’s detection capability for obstacles (vehicles, pedestrians):

(23) $$AP=\int_{0}^{1}p\left(r\right)dr$$

where *P*(*r*) denotes the precision-recall curve.

Mean Average Precision (*mAP*): The *mAP* is computed as the mean of *AP* over all object categories:

(24) $$mAP=\frac{1}{N}\sum_{i=1}^{N}AP_{i}$$

where *N* is the number of detection categories in the multi-class detection task.

Fusion Consistency Score (*FCS*): This metric quantifies the effectiveness of multimodal (LiDAR and camera) information fusion. The target matching rate between heterogeneous sensors is defined as:

(25) $$FCS=\frac{\sum_{k=1}^{M}\theta \left(L_{K}\cap V_{K}\right)}{\max\left(M_{L},M_{V}\right)}$$

where *L_k_* and *V_k_* denote the detection results from the LiDAR and vision branches for the *k*-th target, respectively, *M* is the total number of targets, and θ(⋅) is the intersection indicator function.

(2) Trajectory Prediction and Control Metrics

To evaluate the trajectory prediction and planning capabilities of the end-to-end model, the following core metrics are defined.

(26) $$ADE=\frac{1}{T\cdot N}\sum_{i=1}^{N}\sum_{t=1}^{T}\sqrt{{\left(x_{t}^{i}-x_{t1}^{i}\right)}^{2}+{\left(y_{t}^{i}-y_{t1}^{i}\right)}^{2}}$$

Average Displacement Error (*ADE*): *ADE* measures the average Euclidean distance between the predicted trajectory points and the ground-truth trajectory points across all time steps, reflecting the overall tracking accuracy over the entire prediction horizon. A lower value indicates better trajectory fitting:

Where $\left(x_{t}^{i},y_{t}^{i}\right)$ and $\left(x_{t1}^{i},y_{t1}^{i}\right)$ denote the ground-truth and predicted trajectory points at timestamp *t* for the *i*-th sample, respectively, *T* is the prediction horizon, and *N* is the number of test samples.

Final Displacement Error (*FDE*): *FDE* measures the accuracy of the final predicted trajectory point, which is particularly critical for decision-making in scenarios such as deceleration, stopping, or obstacle avoidance. It is defined as the Euclidean distance between the predicted endpoint and the ground-truth endpoint:

(27) $$FDE=\frac{1}{N}\sum_{i=1}^{N}\sqrt{{\left(x_{t}^{i}-x_{t1}^{i}\right)}^{2}+{\left(y_{t}^{i}-y_{t1}^{i}\right)}^{2}}$$

Mean Squared Error (*MSE*): *MSE* directly reflects the squared average error between the predicted values and the ground-truth values:

(28) $$MSE=\frac{1}{n}{\sum_{i=1}^{n}\left(y_{i}-{\hat{y}}_{i}\right)}^{2}$$

Root Mean Squared Error (*RMSE*): *RMSE* is the square root of *MSE*, which restores the original units and provides a more interpretable measure of error magnitude:

(29) $$RMSE=\sqrt{MSE}$$

(3) System Safety Metrics

Based on the safety distance model, we evaluate potential risks during testing and compute the Collision Risk Probability (*CRP*):

(30) $$P_{collision}=\frac{N_{risk}}{N_{total}}$$

where *P_collision_* denotes the collision risk probability representing the proportion of potential collision risks during testing, *N_risk_* is the number of times the safe distance is exceeded, and *N_total_* is the total number of test steps or time steps.

In dynamic environments, safe distance and collision risk probability may vary over time. To account for this, we introduce a time window *T* and compute the collision risk probability within the window:

(31) $$P_{collision}\left(T\right)=\frac{\sum_{t=1}^{T}\left(d\left(t\right)<d_{safe}\left(t\right)\right)}{T}$$

where *d*(*t*) is the distance at time *t*.

(4) Complementary Performance Metrics

To comprehensively evaluate the overall driving performance, we introduce the following complementary metrics:

Route Completion (%): The percentage of the total route distance that the vehicle successfully traverses within the allocated time limit. A higher value indicates better navigation capability.

Collisions: The number of collision events with other vehicles, pedestrians, or static obstacles during the entire test run.

False Braking Count: The number of unnecessary braking events caused by false positive detections or overly conservative planning. A lower value indicates more comfortable and human-like driving behavior.

Completion Time (s): The total time taken to complete the route. This metric reflects the efficiency of the driving policy.

#### 4.3.2. Model Performance Comparison and Analysis

In this study, a multi-dimensional comparative experimental setup is constructed to systematically validate the performance advantages of the proposed unmanned ground vehicle autonomous driving system in complex urban scenarios. The experimental group comprises four driving strategies: a hybrid planning model based on traditional autonomous driving algorithms, a vision-only reinforcement learning model, a professional human driver benchmark, and the proposed multimodal model. An evaluation system is established using multiple core metrics, focusing on four dimensions: decision-making timeliness, safety fault tolerance, trajectory planning quality, and energy economy.

- Hybrid planning model based on traditional autonomous driving algorithms: The Hybrid MPC algorithm is adopted, in which the upper layer generates a right-of-way distribution map using real-time semantic segmentation (OpenCV), and the lower layer performs trajectory planning using nonlinear model predictive control (NMPC).
- Vision-only reinforcement learning model: A typical DQN + LSTM framework is employed. The visual encoding layer uses a CNN architecture, and the temporal modeling layer uses an LSTM to receive continuous visual features, modeling temporal dependencies through 128-dimensional LSTM units. The reinforcement decision layer feeds the final hidden state of the LSTM into a fully connected network.
- Professional human driver benchmark: A benchmark is constructed by collecting data from professional drivers manually operating the vehicle, which is used to evaluate the intelligence level of the algorithm.

**Figure 20:**
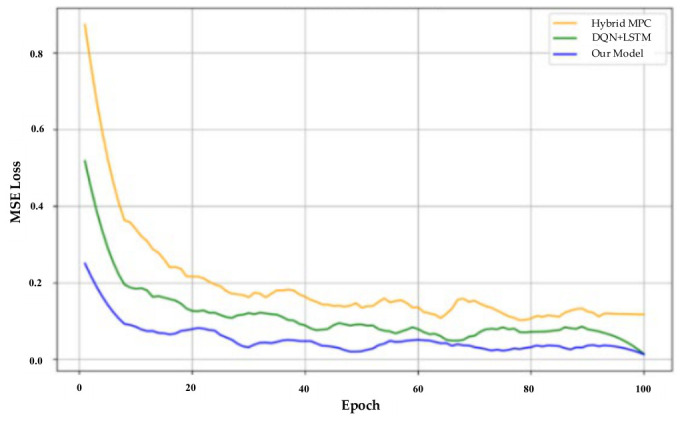
Line chart comparing the convergence speed of different models.

As shown in Figure 20, the training convergence speed of each model is illustrated using the Mean Squared Error (MSE) loss as an indicator. In the initial state, Hybrid MPC exhibits the highest loss value, while the proposed model achieves the lowest loss. As the number of training epochs increases, the MSE losses of all three models show a decreasing trend. Among them, the proposed model converges relatively faster: when the epoch reaches approximately 20, its MSE loss drops to around 0.1; when the epoch reaches 100, its MSE loss is the lowest, approaching zero. This indicates that the proposed model performs best in reducing MSE loss and achieves higher prediction accuracy. Table 6 compares the comprehensive performance of the four driving algorithms across four clearly defined metrics: Route Completion (%), Collisions, False Braking Count, and Completion Time.

The experimental results show that the multi-head attention distillation mechanism designed in this study exhibits significant advantages across all evaluated metrics. In multiple complex traffic scenarios, the proposed algorithm efficiently completes the planned routes, achieving excellent performance on key indicators such as route completion rate (97.26%) and collision frequency (0.2 collisions per test run). The control performance of the algorithm approaches that of manual operation (99.33% route completion, 0.5 collisions), demonstrating a high level of intelligence and effectively meeting the intended design goals of the algorithm.

#### 4.3.3. Model Testing and Analysis

In this study, the model is trained under the knowledge distillation paradigm using a real-world multimodal driving trajectory dataset. During training, an adaptive stochastic gradient descent optimizer combined with a KL divergence loss function is introduced, and a dynamic learning rate scheduling strategy is constructed to ensure progressive parameter convergence between the teacher and student networks during feature sharing. At the network architecture level, a channel-weighted attention transfer module is designed to convey the spatiotemporal correlation features of the teacher network to the lightweight student network in a differentiable manner.

**Figure 21:**
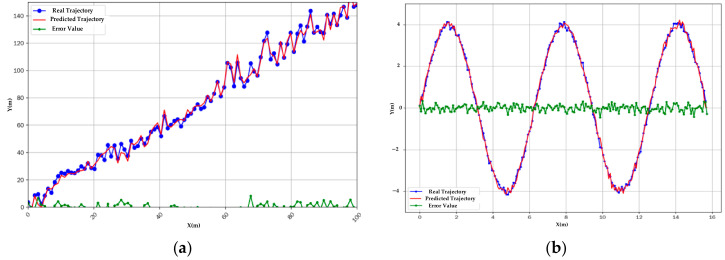
Comparison of trajectory data and prediction results. (**a**) Straight trajectory; (**b**) S-shaped trajectory.

To verify the accuracy of trajectory prediction, a multimodal trajectory validation framework is built: the probability distribution output by the decoder is structurally sampled, and the optimal path is extracted through linear algebraic transformations; then the continuous trajectory function is reconstructed via numerical integration. Figure 21 presents the visual comparison of trajectories. The ground-truth trajectory (blue curve) reflects the actual path of the unmanned ground vehicle, exhibiting high stochasticity and complexity, while the model prediction (red curve) is the trajectory predicted by the proposed distillation model.

Analysis of the driving data in Figure 21a shows that when the x-coordinate exceeds 80 m, the ground-truth trajectory exhibits increased fluctuation with a jagged pattern, indicating that the unmanned ground vehicle is operating under complex and rapidly changing trajectory conditions, which challenges the model’s prediction capability. Nevertheless, the predicted values still follow the overall trend, with only small deviations from the ground truth in local fluctuation details. Figure 21b demonstrates that the model’s tracking performance remains very stable at curves; the Euclidean deviation between the ground-truth trajectory and the predicted trajectory is consistently controlled within 0.5 m, validating the stability of the prediction results. Overall, the ground-truth trajectory and the predicted values show good consistency in their general trends, indicating that the proposed distillation model can capture the variation patterns of autonomous driving trajectories to a certain extent.

**Figure 22:**
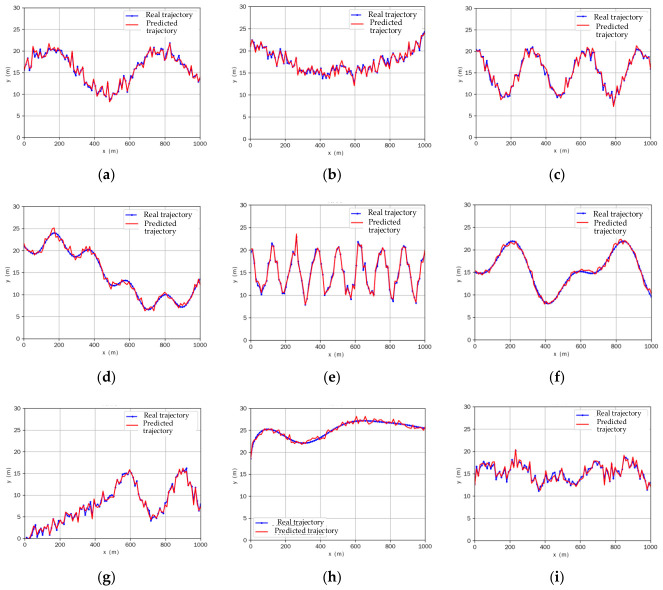
Visualization of trajectory prediction: (**a**) Route 1; (**b**) Route 2; (**c**) Route 3; (**d**) Route 4; (**e**) Route 5; (**f**) Route 6; (**g**) Route 7; (**h**) Route 8; (**i**) Route 9.

To further evaluate the effectiveness of the proposed algorithm in trajectory prediction, multiple sets of experiments are conducted. Figure 22 presents the visualization of nine sets of prediction results. It can be observed that the trajectories predicted by the proposed algorithm are very close to the corresponding ground-truth trajectories, exhibiting similar trends and characteristics, which demonstrates the consistently high accuracy of the proposed algorithm in trajectory prediction.

#### 4.3.4. Experimental Validation Analysis

(1) Vectorized Scene Visualization

**Figure 23:**
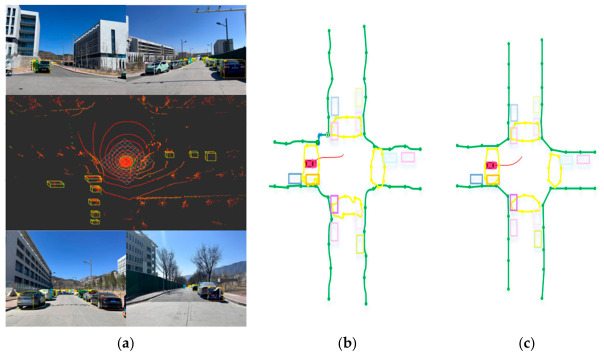
Comparison of vectorized results in a straight-driving scenario. (**a**) Experimental scene; (**b**) Vectorized scene obtained by traditional distillation algorithm; (**c**) Vectorized scene obtained by the proposed multi-head distillation algorithm.

**Figure 24:**
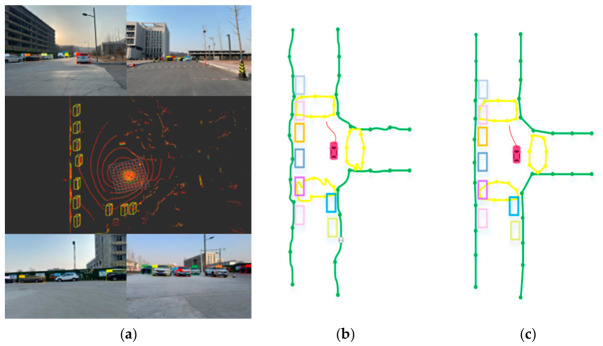
Comparison of vectorized results in a turning intersection scenario. (**a**) Experimental scene; (**b**) Vectorized scene obtained by traditional distillation algorithm; (**c**) Vectorized scene obtained by the proposed multi-head distillation algorithm.

As shown in Figure 23 and Figure 24, the unmanned ground vehicle generates vectorized scenes through the multimodal fusion network based on data collected by multiple sensors. The yellow lines indicate the predicted trajectories. Specifically, Figure 23b and Figure 24b shows the vectorized scene representation obtained using a traditional knowledge distillation algorithm, while Figure 23c and Figure 24c presents that of the proposed algorithm. A comparison reveals that the proposed model achieves significant improvements in both environmental perception and trajectory prediction performance.

(2) Visualization of Control and Planning Experimental Results

**Figure 25:**
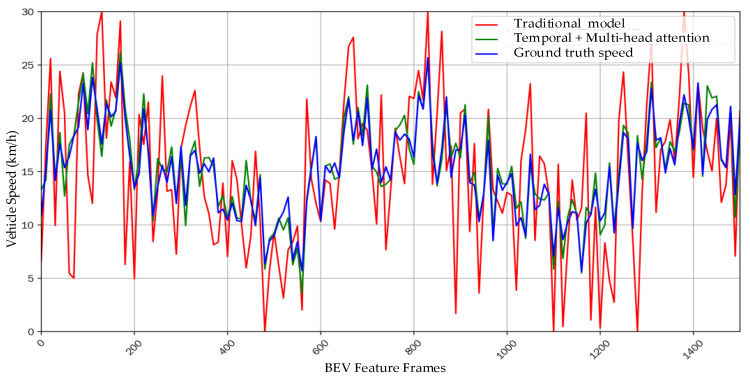
Comparison of speed curves for the UGV with multimodal perception.

**Figure 26:**
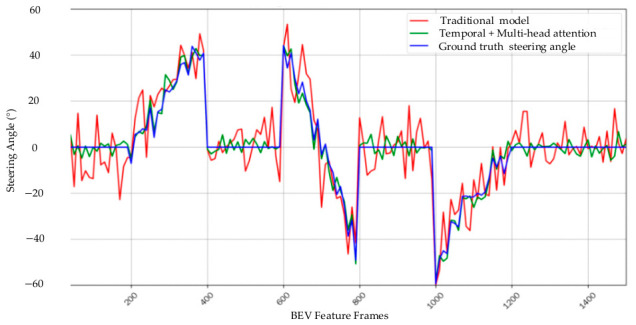
Comparison of steering angle curves for the UGV with multimodal perception.

To verify the feasibility of the proposed control and planning algorithm, experiments were conducted with the unmanned ground vehicle under highly dynamic traffic interactions and nonlinear complex driving scenarios. Figure 25 and Figure 26 present the vehicle speed curve and the steering angle curve, respectively.

As shown in Figure 25 (vehicle speed prediction curves), under nonlinear maneuvering and highly dynamic traffic interaction conditions, the predictions of the traditional knowledge distillation model (red curve) exhibit significant deviations from the actual vehicle speed (blue curve). This is reflected in large prediction errors during speed transients (e.g., acceleration/deceleration switching points) over multiple time intervals, revealing the limitations of the traditional model in multimodal temporal feature extraction and dynamic response fitting. In contrast, the improved model incorporating temporal modeling and a multi-head attention mechanism (green curve) achieves a better fit to the actual speed, indicating that the architecture effectively enhances the decoding capability for complex speed profiles through a spatiotemporal attention weight allocation mechanism, enabling precise alignment of both global and local dynamic features.

Figure 26 (steering angle curve comparison) further verifies the advantages of the improved model. The traditional model (red curve) exhibits both lag and overshoot in its predictions during rapid steering angle adjustments, indicating insufficient modeling of nonlinear steering dynamics. After introducing the temporal-attention distillation mechanism (green curve), the model not only suppresses high-frequency oscillations but also responds quickly to abrupt steering events. This optimization significantly enhances the stability control capability of the unmanned ground vehicle in complex curved roads and obstacle avoidance scenarios.

In Table 7, the MSE loss of the proposed distillation model is the sum of the wheel angle loss and the speed loss, with a weight ratio of 1:1. As shown in Table 7, the proposed model achieves lower MSE loss values on the training, validation, and test sets than the traditional knowledge distillation model, indicating better convergence performance.

To evaluate the prediction performance of the wheel steering angle, Table 8 presents the root mean square error (RMSE) comparison between the proposed model and the traditional knowledge distillation model. The results show that the proposed model achieves a smaller RMSE value, indicating that the knowledge distillation model incorporating temporal and multi-head attention mechanisms can effectively improve the prediction of the wheel steering angle and further enhance prediction accuracy.

The knowledge distillation architecture that integrates temporal modeling and multi-head attention mechanisms simultaneously enhances the sensitivity of speed prediction (with a low mean RMSE) and the smoothness of steering angle estimation through a multi-scale spatiotemporal feature fusion strategy. This provides a perception-decision closed-loop guarantee that combines both accuracy and stability for unmanned ground vehicle motion planning in highly dynamic urban scenarios.

A noticeable disparity exists between our open-loop collision rate (15.2%) and closed-loop real-world performance (route completion 97.26%, decision score 96.8). This discrepancy reflects the fundamental difference between the two evaluation protocols. In open-loop evaluation, the model predicts trajectories at each timestamp independently without feedback, and errors accumulate over the horizon. In closed-loop operation, the model continuously replans every 50 ms, correcting errors at each step. The 0.5 m Euclidean deviation reported in real-world tests is the maximum deviation of the continuously updated executed trajectory, whereas the ADE (0.88 m) and FDE (1.32 m) are average errors of open-loop predictions. The values are not directly comparable and serve different evaluation purposes.

## 5. Conclusions

The main contributions of this paper are summarized as follows:

- A multimodal collaborative environment perception solution is proposed. BEVFormer transforms image features into BEV space; a sparse 3D convolutional network hierarchically extracts LiDAR features; and a Transformer-based cross-attention fusion network aligns these features to form a global environmental representation. Experimental results show that the proposed solution improves object detection accuracy by 36.4% in real-world scenarios compared with the unimodal (image-only) baseline.
- An end-to-end decision-making system based on multimodal fusion and multi-head distillation attention is constructed. The trajectory prediction module incorporates a multi-head distillation attention mechanism with dynamic modality weight allocation and progressive teacher-student training. The control and planning module adopts vectorized scene modeling to convert road topology and dynamic obstacles into structured feature vectors. NuScenes results demonstrate that the proposed method reduces ADE and FDE by 4.34% and 10.81%, lowers the collision rate to 15.2%, and cuts control latency to 46 ms.
- A multi-sensor UGV platform is built for end-to-end multimodal autonomous driving. Experimental results show that the proposed multimodal model reduces trajectory prediction MSE by 51.5% and maintains Euclidean deviation within 0.5 m. With the multi-head attention distillation mechanism, the system significantly reduces collision rate and improves feature decoupling efficiency.

This study still requires further research in (1) automating the distillation training process through online distillation techniques and (2) developing formal verification toolchains to enhance interpretability and safety of the distillation-rule hybrid strategy.

In summary, this study provides a systematic solution for end-to-end autonomous driving based on multimodal fusion for UGVs, driving the technology toward high reliability, high efficiency, and strong generalization.

## Figures and Tables

**Table 1:** Task allocation of attention heads in the teacher model.

Head Index	Focus Dimension	Function Description
1–2	Temporal continuity	Predict temporal changes in target acceleration and steering angle.
3–4	Spatial obstacle interaction	Detect collision risks from surrounding vehicles and pedestrians.
5–6	Social group dynamics	Analyze crowd movement trends and avoidance intentions.
7–8	Cross-modal consistency verification	Align visual and LiDAR structural features.

**Table 2:** Trajectory data information.

Field	Description
TIMESTAMP	Absolute timestamp in seconds
TRACK_ID	ID of the traffic participant, unique for each vehicle
OBJECT_TYPE	Traffic participant types include AV (ego vehicle), AGENT (target agent), and OTHERS (other agents). OTHERS does not differentiate among vehicles, pedestrians, etc.
X	X coordinate in the global coordinate system
Y	Y coordinate in the global coordinate system

**Table 3:** Comparison of trajectory prediction performance.

Method	ADE (m)	FDE (m)	Collision Rate (%)
Uncompressed teacher model	0.78	1.25	14.3
Traditional knowledge distillation model	0.92	1.48	19.8
Lightweight student model without distillation	1.05	2.03	22.4
Multimodal multi-head attention distillation model	0.88	1.32	15.2

**Table 4:** Comparison of computational efficiency before and after distillation.

Method	Params (M)	FLOPs (G)	Latency (ms)
Uncompressed teacher model	125.6	70.3	92
Traditional knowledge distillation model	36	20	43
Lightweight student model without distillation	34	19	42
Multimodal multi-head attention distillation model	42.6	32.8	46

**Table 5:** Ablation experiment results.

*L_traj_*	*L_attn_*	*L_feat_*	ADE (m)	FDE (m)
√	--	√	0.95	1.50
√	√	--	0.97	1.58
√	--	--	1.05	1.72
--	√	√	1.24	2.01
√	√	√	0.88	1.32

**Table 6:** Algorithm performance comparison.

Method	Route Completion (%)	Collisions	False Braking Count	Completion Time (s)
Hybrid MPC	60.99	0.6	1.5	420
DQN + LSTM	65.22	0.8	3.2	450
Human Driving	99.33	0.5	0.8	400
Ours	97.26	0.2	0.6	408

**Table 7:** Comparison of model MSE.

Model	Training Set	Validation Set	Test Set
Traditional knowledge distillation	0.078	0.088	0.095
Temporal + Multi-head attention	0.039	0.051	0.046

**Table 8:** Comparison of RMSE for wheel steering angle.

Model	RMSE
Traditional knowledge distillation	0.236
Temporal + Multi-head attention	0.098

## Data Availability

The original contributions presented in the study are included in the article, further inquiries can be directed to the corresponding author.

## Abbreviations

The following abbreviations are used in this manuscript:

MPC	Model Predictive Control
NMPC	Nonlinear Model Predictive Control
DQN	Deep Q-Network
KL	Kullback–Leibler
mAP	mean Average Precision
LSTM	Long Short-Term Memory
NDS	NuScenes Detection Score
MLP	Multi-Layer Perceptron
CUDA	Compute Unified Device Architecture
cuDNN	CUDA Deep Neural Network library
VAD	Vectorized Autonomous Driving
TV	Target Vehicle
NV	Neighboring Vehicle
